# The Host-Dependent Interaction of α-Importins with Influenza PB2 Polymerase Subunit Is Required for Virus RNA Replication

**DOI:** 10.1371/journal.pone.0003904

**Published:** 2008-12-10

**Authors:** Patricia Resa-Infante, Núria Jorba, Noelia Zamarreño, Yolanda Fernández, Silvia Juárez, Juan Ortín

**Affiliations:** 1 Centro Nacional de Biotecnología (CSIC) Darwin 3, Campus de Cantoblanco, Madrid, Spain; 2 CIBER de Enfermedades Respiratorias, Mallorca, Illes Balears; Yonsei University, Republic of Korea

## Abstract

The influenza virus polymerase is formed by the PB1, PB2 and PA subunits and is required for virus transcription and replication in the nucleus of infected cells. As PB2 is a relevant host-range determinant we expressed a TAP-tagged PB2 in human cells and isolated intracellular complexes. Alpha-importin was identified as a PB2-associated factor by proteomic analyses. To study the relevance of this interaction for virus replication we mutated the PB2 NLS and analysed the phenotype of mutant subunits, polymerase complexes and RNPs. While mutant PB2 proteins showed reduced nuclear accumulation, they formed polymerase complexes normally when co expressed with PB1 and PA. However, mutant RNPs generated with a viral CAT replicon showed up to hundred-fold reduced CAT accumulation. Rescue of nuclear localisation of mutant PB2 by insertion of an additional SV40 TAg-derived NLS did not revert the mutant phenotype of RNPs. Furthermore, determination of recombinant RNP accumulation in vivo indicated that PB2 NLS mutations drastically reduced virus RNA replication. These results indicate that, above and beyond its role in nuclear accumulation, PB2 interaction with α-importins is required for virus RNA replication. To ascertain whether PB2-α-importin binding could contribute to the adaptation of H5N1 avian viruses to man, their association in vivo was determined. Human alpha importin isoforms associated efficiently to PB2 protein of an H3N2 human virus but bound to diminished and variable extents to PB2 from H5N1 avian or human strains, suggesting that the function of alpha importin during RNA replication is important for the adaptation of avian viruses to the human host.

## Introduction

Transcription and replication of influenza A virus is carried out in the nucleus of the infected cells by each of the eight ribonucleoprotein particles (RNPs) that constitute their genome (reviewed in [1]–[3]). Each RNP contains one single-stranded RNA segment encapsidated by binding to nucleoprotein (NP) monomers and the polymerase complex [4], a heterotrimer containing the PB1, PB2 and PA subunits. Within the RNP, the viral RNA polymerase is the enzyme responsible for transcription and replication. The PB1 subunit is responsible for the polymerase and endonuclease activities, PB2 binds the cap structure of cellular pre-mRNAs and PA is a phosphoprotein with protease activity involved in RNA replication (reviewed in [1], [2]). These subunits form a tight and stable complex whose low-resolution structure has been determined by electron microscopy and image processing [5], [6]. The PB1 subunit is the core of the complex and interacts with both PB2 and PA, whereas no interaction between PB2 and PA proteins has been described. Each polymerase subunit can be translocated to the nucleus as they contain nuclear localisation signals (NLSs) [7]–[9] and the precise structure of the PB2 NLS has recently been determined by co-crystal formation with importin α5 [10]. However, the intracellular site and the pathway for complex formation are not clear at present. It has been proposed that the PB1-PA dimer would be formed in the cytoplasm and transported to the nucleus whereas PB2 would be transported independently [11], [12]. The PB1-PA dimer and PB2 would then form the heterotrimer in the nucleus, as supported by in vitro assembly reactions [13]. Alternative proposals are based on the formation of Hsp90 complexes with PB1 and PB2 and suggest the formation of PB1-PB2 and PB1-PA dimers and their co-transport to the nucleus [14], [15].

In this report we have studied the role in the infection of influenza PB2 subunit by proteomic analysis of intracellular complexes containing PB2 and mutation of the recently described NLS [10]. We found α-importins as PB2-associated host factors and showed that they bind efficiently to PB2 derived from a human virus but associate to a diminished and isoform-specific way to PB2 from H5N1 avian viruses. Although polymerase complex formation was not affected by mutation of PB2 NLS, RNP activity was essentially abolished. Restoring nuclear import by ectopic addition of a SV40 TAg NLS did not rescue the RNP biological activity, suggesting that PB2 interaction with α-importin is required for the biological activity of influenza polymerase, in addition to its role in protein transport. Furthermore, mutation of PB2 NLS abrogated the capacity of recombinant RNPs to accumulate in vivo, indicating that the interaction of importin α with the natural NLS in PB2 is necessary for viral RNA replication.

## Results

### Importins α associate in vivo with the influenza polymerase PB2 subunit

**Figure 1:**
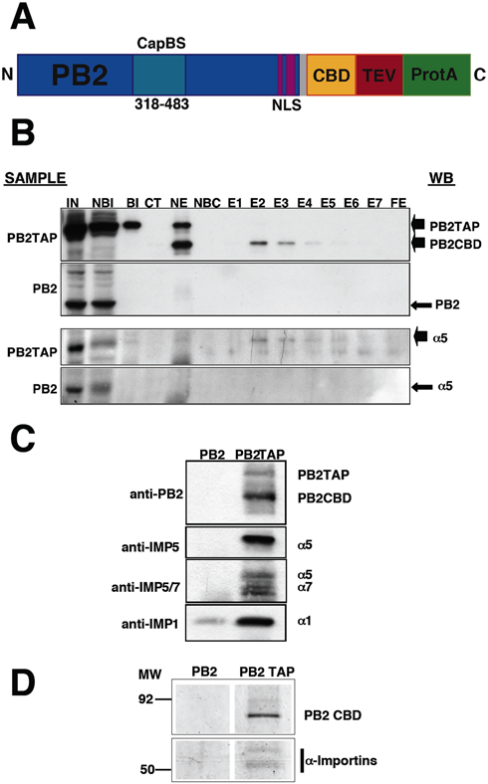
Purification of PB2-containing intracellular complexes. (A) Diagram of the PB2TAP recombinant protein. The structure of the C-terminal TAP tag is shown, with the calmodulin-binding domain (CBD), the TEV cleavage site (TEV) and the IgG-binding domain (ProtA). Also marked are the cap-binding domain (CapBS) [41], and the NLS [10]. (B) Analysis of the purification of recombinant PB2TAP or control PB2 protein by Western-blot. Also shown is the co-purification of α-importin using anti-importin antibodies. The purification fractions analysed were as follows: Input extract (IN), material not bound to IgG (NBI), material bound to IgG (BI), material cleaved with TEV protease (CT), material not eluted from IgG (NE), material not bound to calmodulin-agarose (NBC), elutions from calmodulin-agarose (E1–E7) and material not eluted from calmodulin-agarose (FE). The material used for each purification is indicated on the left (Sample), whereas the position of the Western-blot signals (WB) is shown to the right. (C) Identification of α-importin isoforms associated to PB2. The proteins eluted with TEV protease from the IgG-Sepharose column were analysed by Western-blot with antibodies specific for various forms of α-importin. The antibodies used are indicated to the left whereas the position of the Western-blot signals is shown to the right. (D) Composition of polymerase subunits PB2-associated proteins. Complexes containing PB2 were purified from PB2-TAP- (PB2-TAP) or PB2-transfected cells (PB2), using the TAP approach. Aliquots of the samples after two affinity chromatography steps were analysed by polyacrylamide gel electrophoresis and silver staining. The position of molecular weight markers is indicated to the left. Only the relevant sections of the gel are shown.

To determine the human host factors that associate in vivo with the PB2 subunit of the influenza RNA polymerase we expressed a TAP-tagged version of the protein (Fig. 1A) by transfection of human cells and purified the intracellular complexes formed in vivo by tandem affinity chromatography as described [16]. As a control, untagged PB2 was expressed and purified in parallel. MS-MS analysis of the purified complexes identified the presence of importin α5, in addition to the purified PB2 subunit (Tables S1 and S2). To verify biochemically these results, the purified PB2-containing complexes were analysed by Western-blot with α-importin-specific antibodies. The results presented in Fig. 1B, C show the co-purification of importin α5 with PB2 and the in vivo association of importins α1, α5 and α7 with PB2. These results are consistent with the co-crystallisation of importin α5 with the NLS-containing C-terminal domain of PB2 protein [10] and the co-localisation of importin α1 with PB2 in influenza virus-infected cells [17]. The accepted mechanism for nuclear import implies that importin-cargo interaction is transient and is eliminated once the nuclear envelope barrier is overcome. The fact that it is possible to isolate stable intracellular complexes of PB2 and importin α (Fig. 1D) would suggest that such interaction might have a biological role other than the expected for PB2 nuclear import.

### Mutations in the NLS of PB2 do not alter polymerase complex formation but abolish the activity of viral RNPs

**Figure 2:**
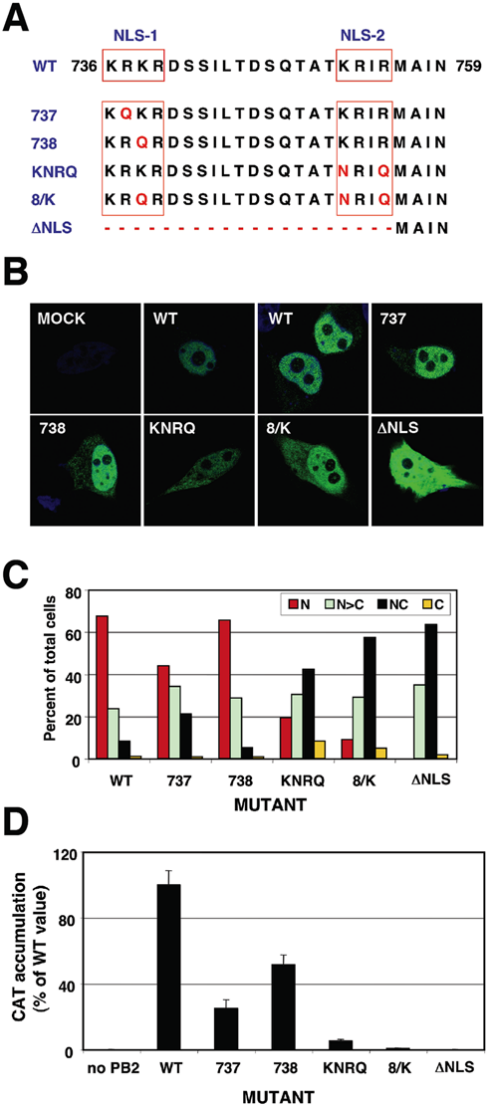
Phenotype of PB2 mutants with altered NLS. (A) Sequence of the PB2 NLS located close to the C-terminus. The position of the two regions of the bipartite NLS (NLS-1 and NLS-2) is indicated, as well as the mutations present in mutants 737, 738, KNRQ, 8/K and ΔNLS. (B) Representative intracellular localisations of wt and mutant PB2 proteins. Central optical sections are presented of HeLa cells mock-transfected (MOCK), transfected with wt PB2 (WT) or with each of the mutant PB2 proteins. Nuclei were stained with DAPI (blue) and PB2 was stained with anti-PB2 monoclonal antibody and goat anti-mouse IgG coupled with Alexa 488 (green). (C) Quantitative estimation of the localisation of wt or mutant PB2 proteins. Around 100 individual cells were scored as: N, exclusively nuclear; N>C, nuclei more stained than the cytoplasm; NC, equal nuclear and cytoplasmic staining or C, exclusively cytoplasmic staining. (D) Biological activity of recombinant RNPs with mutated PB2 proteins. Cultures of HEK293T cells were co-transfected with plasmids expressing PB1, PA, NP and either wt or mutant PB2, as well as a plasmid encoding CAT gene in negative polarity, flanked by the non-translated regions of influenza NS segment. Plasmid expressing PB2 was omitted as a negative control. At 20 hours post-transfection total cell extracts were prepared and CAT protein was determined by ELISA. The averages and standard deviations of 3–9 independent experiments are shown.

**Figure 3:**
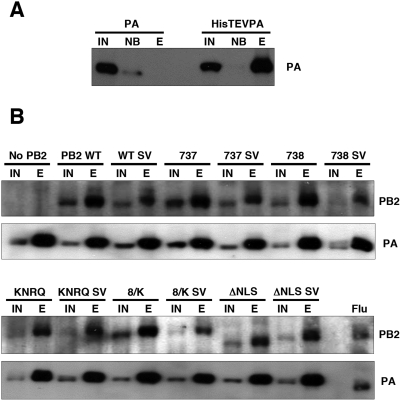
Polymerase complex formation of wt and mutant PB2 proteins. Cultures of HEK293T cells were co-transfected with plasmids expressing PB1, HisTEVPA or PA as a control, and either wt or mutant PB2. PB2 was omitted as a control. Total cell extracts were prepared and analysed by pull-down with ^2+^Ni-NTA-agarose and Western-blot using anti-PB2 and anti-PA monoclonal antibodies. (A). Controls for the pull-down experiment. (B). Analysis of PB2 mutants. IN: Input extract. NB: Not bound to the resin. E: Eluted fractions. SV denotes that an additional NLS derived from SV40 TAg has been inserted. An extract obtained from influenza virus-infected cells was used as mobility marker (Flu).

To study the possible functions of PB2-importin α interactions we generated PB2 mutants altered in the bipartite NLS. Mutants in NLS-1 (R737Q –mutant 737- or K738Q –mutant 738-), in NLS-2 (K752N+R755Q –mutant KNRQ-) or in both regions (K738Q+K752N+R755Q -mutant 8/K-) were generated. These mutations were described previously on the basis of the co-crystal structure of PB2 NLS domain bound to alpha importin and progressively decreased the interaction of PB2 with the importin [10]. In addition, a deletion mutant in which the complete NLS was eliminated was also produced –mutant ΔNLS- (Fig. 2A). After verifying that all mutant PB2 proteins were expressed correctly (Fig. S1), their nuclear import in HeLa cells was tested by indirect immunofluorescence using PB2-specific antibodies. Representative images are presented in Fig. 2B and a summary of the phenotypes is shown in Fig. 2C. The results are consistent with those reported for the mutant C-terminal domain of PB2 fused to GFP [10] and indicate that nuclear accumulation of untagged PB2 protein is slightly affected by single-point mutations in NLS1 and more inhibited by a double mutation in NLS2. Thus, only slight PB2 cytoplasmic staining was apparent in the 737 or 738 mutants, while mutants KNRQ and 8/K had similar concentrations of PB2 in the nucleus and the cytoplasm. Even mutant ΔNLS showed some nuclear staining. Similar phenotypes were observed in HEK293T cells (Fig. S2). The biological activity of these mutants was first tested in a RNP replication system using a CAT gene-containing viral replicon. Cells were transfected with plasmids encoding each of the polymerase subunits, the NP and the recombinant replicon. Either wt or mutant PB2-encoding plasmids were used, while PB2 plasmid was omitted as a negative control. The CAT protein accumulation was used as a measure of the combined replication-transcription activity of each recombinant RNP. The results obtained are presented in Fig. 2D and show that the NLS mutations in PB2 lead to a much stronger phenotype than that expected from the intracellular localisation of the protein. Thus, mutants 737 and 738, which show a localisation hardly distinguishable from wt PB2 (Fig. 2B), led to RNPs 2–4 times less active than wt RNPs. On the other hand, mutants KNRQ and 8/K, which still show considerable accumulation of PB2 in the nucleus (Fig. 2B), gave rise to RNPs 10–100 times less active than wt. In addition, mutant ΔNLS, which was not completely excluded from the nucleus, was completely inactive. These results indicated that there is a correlation between the progressive reduction in mutant PB2 domain interaction with importin alpha [10] and a decrease in the activity of the mutant RNPs. However, the much stronger activity phenotype suggested that the defect in PB2 intracellular localisation is not the only cause for the reduction in mutant RNP activity and led us to test other alternatives. A naïve explanation would be that the polymerase complex is not formed. Hence, we tested this possibility by cotransfection of plasmids expressing PB1, HisTEVPA and either wt or mutant PB2, and pull-down with Ni-NTA-agarose. Complex formation was ascertained by Western-blot with anti-PB2 antibodies and is shown in Fig. 3. No significant differences were observed in the capacity of mutant or wt PB2 proteins to produce polymerase complexes.

### Insertion of an ectopic NLS does not rescue the activity of RNPs generated with NLS PB2 mutants

**Figure 4:**
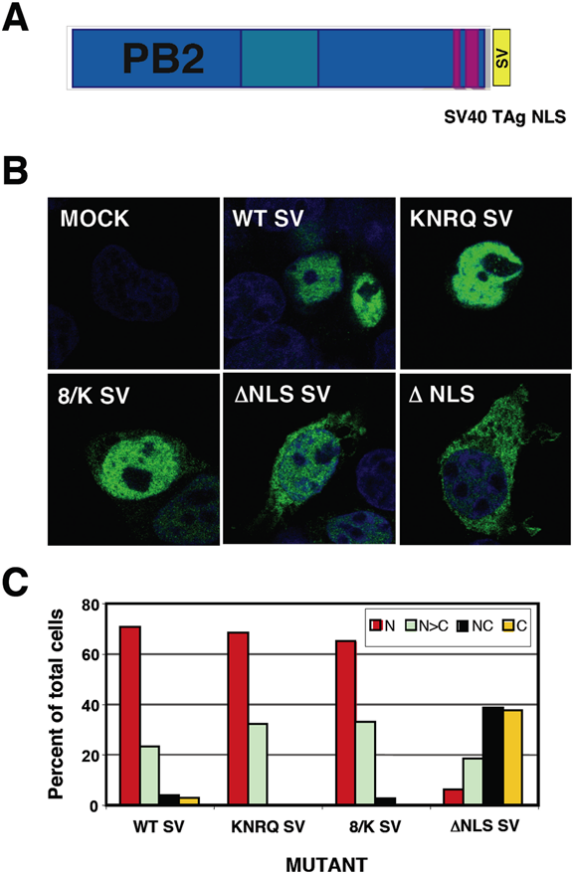
Intracellular localisation of mutated PB2 proteins containing an ectopic NLS. (A) Diagram showing the structure of PB2 protein containing a SV40 TAg NLS at its C-terminus. (B) Representative phenotypes of the intracellular localisation of wt and mutant PB2 proteins. Cultures of HEK293T cells were transfected with plasmids expressing either wt or mutant PB2 proteins containing an additional NLS signal at the protein C-terminus. Central optical sections are presented of mock-transfected (MOCK), transfected with wt PB2 (WT) or with each of the mutant PB2 proteins. The phenotype of the ΔNLS mutant lacking the SV40 TAg NLS is also shown. Nuclei were stained with DAPI (blue) and PB2 was stained with anti-PB2 monoclonal antibody and goat anti-mouse IgG coupled with Alexa 488 (green) (C) Quantitative estimation of the localisation of wt or mutant PB2 proteins was performed as indicated in Fig. 2C.

**Figure 5:**
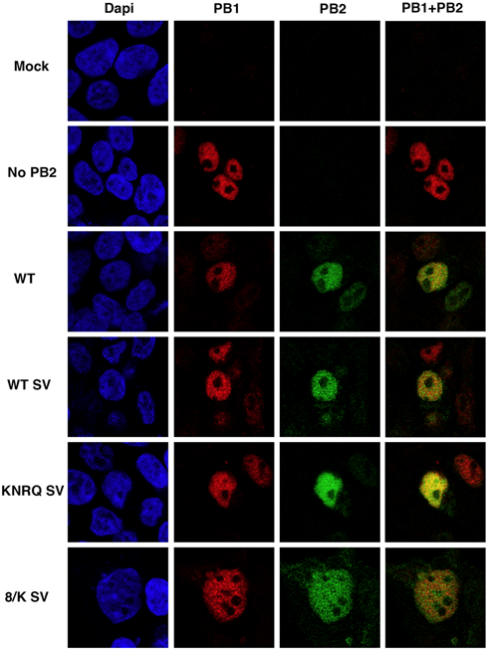
Intracellular localisation of wt and mutant PB2 proteins in cells co-expressing the polymerase subunits. Cultures of HEK293T cells were co-transfected with plasmids expressing PB1, PA and either wt or mutant PB2 containing an additional TAg NLS. The cultures were fixed and analysed by double immunofluorescence using antibodies specific for PB1 and PB2 proteins. The signals for nuclear staining (DAPI –blue-), PB1 protein (PB1 –red-), PB2 protein (PB2 –green-) and the merge of PB1 and PB2 signals are shown for mock-transfected cells (Mock), cells expressing PB1 and PA (No PB2), cells expressing wt polymerase (WT), cells expressing polymerase with PB2-SV protein (WT SV) or cells expressing mutant polymerase containing each of the PB2 mutants with additional TAg NLS (KNRQ SV and 8/K SV).

**Figure 6:**
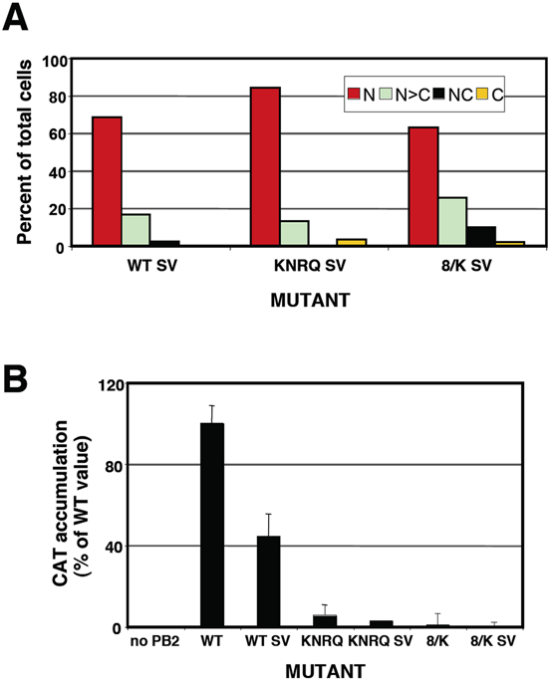
Biological activity of recombinant RNPs with an ectopic NLS in PB2 protein. (A) Quantitative estimation of the localisation of wt or mutant PB2 proteins was performed as indicated in Fig. 2C. (B) Cultures of HEK293T cells were co-transfected with plasmids expressing PB1, PA, NP and either wt or mutant PB2, with or without an additional TAg NLS fused to the C-terminus of the protein. In addition, a plasmid encoding CAT gene in negative polarity, flanked by the non-translated regions of influenza NS segment was co-transfected to provide a viral replicon. Plasmid expressing PB2 was omitted as a negative control. At 20 hours post-transfection total cell extracts were prepared and CAT protein was determined by ELISA. The averages and standard deviations of 3–9 independent experiments are shown.

To distinguish whether the interaction of α-importins to the C-terminal NLS of PB2 is only important for its nuclear accumulation or it rather plays additional roles in the activity of the virus RNPs we tried to rescue the nuclear localisation of the PB2 mutants by the ectopic insertion of an alternative NLS. Thus, a SV40 TAg NLS was inserted at the C-terminus of either wt or each of the most affected PB2 mutants, KNRQ, 8/K and ΔNLS (see Fig. 4A for a diagram), and the localisations of the chimeric proteins were tested by indirect immunofluorescence of transfected cells. As expected, nuclear localisation was maintained in the wt protein and was rescued for the KNRQ and 8/K mutant proteins containing a TAg NLS (Fig. 4B, C). As rescue of ΔNLS mutant was not complete, its phenotype was not analysed further. After verification that the TAg NLS-containing PB2 proteins formed heterotrimeric polymerase complexes efficiently (Fig. 3), we tested the intracellular localisation of wt or mutant PB2 proteins in cells co-expressing the other subunits of the polymerase. Cultures of HEK293T cells were cotransfected with plasmids expressing PB1, PA and either wt or the mutant PB2 proteins with stronger phenotypes (KNRQ SV or 8/K SV). As it has been shown that nuclear transport of PB1-PA and PB2 proteins are independent [11], [13], the cells were analysed by immunofluorescence by double-staining with antibodies specific for PB1 and PB2. Representative images are presented in Fig. 5 and the quantitation of the data appears in Fig. 6A. These results show that both wt and mutant PB2 proteins containing a TAg NLS are localised to the nucleus in cells positive for PB1 staining. Therefore, neither the NLS point mutations nor the insertion of the TAg NLS altered significantly the folding of PB2, as it could correctly form polymerase complex (Fig. 3) and be transported to the nucleus (Figs 5 and 6A).

The activity of each of these rescued mutant RNPs was again tested in the CAT minireplicon assay as described above and the results are presented in Fig. 6B. The insertion of a TAg NLS downstream of wt PB2 led to a 2–3-fold decrease in the accumulation of CAT protein and similar reductions were observed for the KNRQ and 8/K mutants (Fig. 6B). No rescue of CAT expression was observed for RNPs with KNRQ or 8/K mutations in PB2 by insertion of an additional NLS, in spite of an almost complete nuclear localisation of the mutant PB2 proteins. Therefore, we can conclude that the lack of nuclear localisation of PB2 mutants is not the main cause for the lack of RNP activity. Rather, the NLS mutations must affect a step downstream in the infection cycle, probably during RNA replication or transcription.

### Mutation of the PB2 NLS affects viral RNA replication

**Figure 7:**
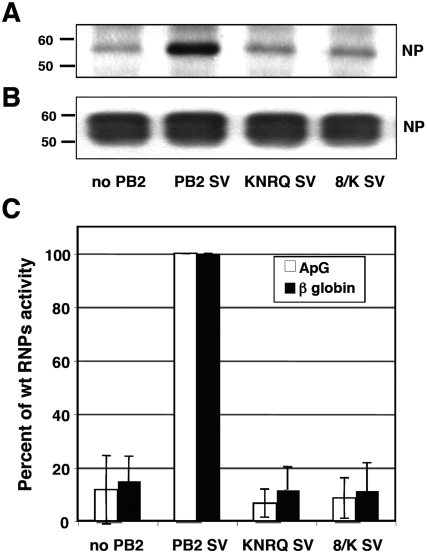
Replication capacity of recombinant RNPs with an ectopic NLS in PB2 protein. HEK293T cells were co-transfected with plasmids expressing either PB1-TAP or PB1, PA, NP and either wt or mutant PB2 containing an additional TAg NLS, as well as a plasmid encoding a viral replicon containing 248 nt [5]. Twenty four hours after transfection, extracts were prepared and progeny RNPs were purified by affinity chromatography on IgG-Sepharose and cleavage with TEV protease. (A) Accumulation of purified RNPs as determined by Western-blot using anti-NP specific antibodies. (B) Total NP present in the cell extracts before purification. (C) Accumulation of purified RNPs as determined by transcription in vitro using ApG (white bars) of β-globin (black bars) as primers. The data presented are the averages and standard deviations from three independent purification experiments.

To determine which step in viral replication requires the interaction of virus polymerase with importin α, we reconstituted in vivo RNPs using as replicon a 248 nt long negative polarity RNA derived from viral RNA 8 [18]. Such RNPs accumulate efficiently in vivo and can be purified biochemically [4], [5]. The cell cultures were transfected with plasmids expressing PA, NP and either wt or mutant PB2 proteins containing an additional TAg NLS. To allow RNP purification, a TAP-tagged PB1 protein was coexpressed [5], [16]; alternatively, untagged PB1 was used as a control. In this way, the progeny RNPs produced in vivo could be bound to IgG-Sepharose and eluted specifically by digestion with TEV protease [16]. In this assay, the amount of purified recombinant RNPs is a measure of their replication in vivo, as any polymerase null mutation abolish the generation of RNPs [19], [20]. The accumulation of viral RNPs was measured by Western-blot with antibodies specific for NP. The results are presented in Fig. 7A, B and show clearly that the accumulation of RNPs in vivo was almost abolished when the KNRQ or 8/K mutations were introduced in PB2 protein. These results were confirmed by determination of the transcriptional activity of the purified RNPs. The average and standard deviation of three independent purification experiments are presented in Fig. 7C, again showing that essentially no in vivo accumulation of RNPs occurred when mutations KNRQ or 8/K were introduced in PB2.

### Strain-dependent interaction of human α-importins to the PB2 subunits of influenza viruses

**Figure 8:**
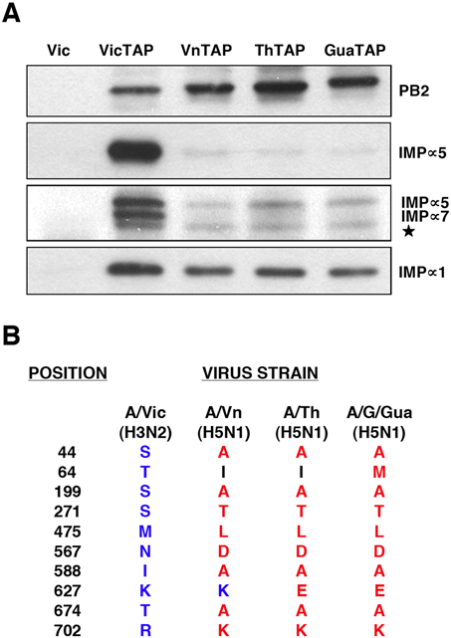
Association of human α-importin isoforms to PB2 protein from various viral strains. The PB2 proteins from either A/Victoria/3/75 (VicTAP), A/Vietnam/1203/04 (VnTAP), A/Thailand/Kan1/04 (ThTAP) or A/Goose/Guandong/1/97 (GuaTAP) strains were TAP-tagged and expressed by transfection into human HEK293T cells. Untagged PB2 from A/Victoria/3/75 (Vic) virus was used as a control. The PB2 protein was purified by binding to IgG-Sepharose and elution with TEV protease. (A) Analysis of the eluates by Western-blot using antisera specific for α-importins α1, α5 and α5/7, as well as anti-PB2. The position of the specific bands is indicated to the right. The star denotes a cross-reaction band of unknown origin. (B) Comparison of the PB2 sequences of the virus strains used, at the positions identified as linked to host-range [26]. No differences were observed among these PB2 proteins at the C-terminal NLS.

The PB2 protein is one of the most relevant viral determinants for avian virus adaptation to human cells [21], [22] and adaptation of an avian virus to mice involves alteration of PB2 interaction with α-importins [17]. These facts, together with the importance of the interaction of PB2 with α-importins for viral replication prompted us to test whether PB2 proteins derived from viruses of different phylogenetic origins would show differential capacities for an stable association to human α-importins. Thus, the PB2 coding regions of A/Goose/Guandong/1/97 (H5N1), an avian strain, and those from A/Vietnam/1203/04 (H5N1) and A/Thailand/Kan1/04 (H5N1), two viruses isolated from sporadic avian influenza cases in humans, were TAP-tagged, expressed in human cells and used to pull-down α-importins. As a positive control, A/Victoria/3/75 (H3N2) PB2 was used, as described in Fig. 1C, whereas untagged PB2 was employed as a negative control. The results are presented in Fig. 8. A much reduced association was observed for PB2 proteins of avian H5N1 viruses, both isolated from avian species or from sporadic cases in man. In addition, the interaction is clearly dependent on the α-importin isoform. Thus, **α**5-importin and **α**7-importin interaction to PB2 from H5N1 viruses was negligible, whereas association of α1-importin was only partly diminished as compared with the signal for the PB2 from a human H3N2 virus. These results indicate that the stable interaction of PB2 with **α**-importins is host-dependent. Furthermore, the reduced co-purification of α-importins, particularly **α**5- and **α**7-importins, with PB2 proteins from H5N1 viruses correlate with their reduced replication capacity in human cells [23], suggesting that this interaction might play a role in the adaptation of avian H5N1 viruses to replication in the human host.

## Discussion

The PB2 subunit of the influenza polymerase is an essential element for virus RNA transcription and replication [20], [24], [25] and, in addition, it has been described as a relevant viral gene in the definition of host-range [21]–[23], [26]–[28]. Hence we set out to purify intracellular complexes containing PB2 protein using the TAP purification approach [29], as performed for the polymerase complex [16]. It was not surprising to identify α-importin among the proteins associated to PB2 (Fig. 1), since a NLS has been identified at its C-terminus [7] and a PB2 C-terminal domain could be co-crystallised with α-importin [10]. However, the stability of the interaction in vivo was unexpected (Fig. 1D) and hence the relevance of the PB2 interaction to α-importins for influenza virus infection was analysed by mutation. In agreement with previous data reported for NLS domain-α-importin interaction, mutation of the NLS resulted in defective nuclear import of mutant PB2 proteins, although none of the mutations led to exclusion of the protein from the nucleus (Fig. 2B) and their capacity to form polymerase complexes in vivo was not altered (Fig. 3). In spite of proper formation of polymerase complexes, the activity of RNPs including NLS mutations in PB2 was progressively reduced, in parallel to the decrease in their ability to interact with α-importin [10], as reflected in the activity of a CAT replicon in vivo (Fig. 2D). Altogether, the results obtained suggested that the interaction of α-importins with the C-terminal NLS in PB2 might serve an additional role, distinct from its normal function for nuclear import, during influenza virus infection. A usual approach to test the relevance of a host factor in a virus infection is to down-regulate its expression by gene silencing and analyse the consequences for the replication of the virus. Such an approach would not be useful here, as down-regulation of α-importins would reduce nuclear accumulation of PB2 (and other cellular and viral proteins) and hence lead to indirect, non-informative phenotypes. As an alternative, we rescued the nuclear localisation of NLS PB2 mutants by insertion of an additional NLS derived from SV40 TAg (Figs. 4, 5) and asked whether the rescued mutants would lead to the recovery of the biological activity of the RNPs in vivo. The results showed clearly that this was not the case (Fig. 6B), indicating that indirectly driven nuclear localisation is not sufficient for RNP activity and suggesting that correct binding of α-importin to the *bona-fide* PB2 NLS is required. The correlation between (i) the import phenotype of the various mutants generated, (ii) their ability to interact in vitro with α-importin [10] and (iii) the reduced activity of the mutant-containing RNPs, makes it very unlikely that the functional defect described here is unrelated to the alterations in PB2-α-importin interaction. It could be argued that insertion of the SV40 TAg NLS itself impairs the activity of the RNPs but this is also unlikely since (i) it only diminishes the activity of wt RNPs by twofold (Fig. 6B) and (ii) insertion of any tags at PB2 C-terminus (His, TAP, GFP) did not alter the activity of RNPs [5], [11].

As PB2 protein is involved in the cap-RNA recognition required for cap-snatching, it might be hypothesized that interaction with α-importin is important for viral transcription initiation. However, purified viral RNPs, either derived from virions or isolated from a recombinant system, are capable of efficient in vitro transcription using capped mRNA as a cap donor [5] (R. Coloma, unpublished results), suggesting that α-importin is not an essential cofactor for viral transcription. Indeed, the experiments reported here revealed that alteration of PB2 NLS abolishes virus RNP accumulation in vivo (Fig. 7). These results are consistent with the transient accumulation of α-importin in the nucleus of infected cells [17] and are easier interpreted by proposing that α-importin acts as a cofactor for virus RNA replication, although we can not exclude that it also plays a role during transcription of progeny RNPs. At least two alternative models can be put forward to explain the requirement of α-importin: either *bona fide* interaction of PB2 with α-importin is necessary for the proper folding of an active viral polymerase complex or it is needed for RNP replication itself. Further experimentation will be required to distinguish between both possibilities.

Our results should also be put in the perspective of the adaptation of avian influenza viruses to mammalian hosts, in particular to man. As indicated above, the PB2 gene is a major player in the ability of avian polymerase complexes to replicate in mammalian hosts [21]–[23], [27], [28] (reviewed in [30]). A series of mutation signatures in PB2 have been identified that accompany avian virus adaptation [26], some of which are located close to the NLS-containing domain in the primary sequence (for example A674T, K702R) (Fig. 8B). Other host-range mutations lay far apart in the sequence of PB2 [26] but it is possible that some of them are spatially close to the α-importin binding site and might alter the interaction with α-importin, as suggested from the co-crystal structure determined recently [10]. Moreover, it has been recently shown that mutation D701N stimulates binding of PB2 to α-importin in human but not in avian cells [17]. The differential interaction of PB2 proteins derived from viruses of avian and human origin with human α-importins (Fig. 8A), is spite of having no differences in the C-terminal NLS, suggests that other regions in the protein might be responsible for the stable association observed. This would be in agreement with the observation that, even in mutant 8/K which contains three mutations affecting both sections of the C-terminal NLS, partial nuclear localisation is detected (Fig. 2B,C). In this context it is worth mentioning that the original description of the PB2 NLS [7] identified an internal region (aa 449–495) whose deletion led to incomplete nuclear targeting of PB2. Although this could be interpreted as result of protein misfolding, it could also reflect the possible relevance of amino acid at position 475. This is one of the most tightly host-linked mutations [26] and is leucine in avian viruses and methionine in human strains (Fig. 8B). As a whole, our results are in agreement with the previous proposal that nuclear transport of the viral polymerase might be a host-dependent step [17] and further extend the implications of host-dependent PB2 interaction with α-importin by showing that such interaction is stable and important for virus RNA replication itself.

## Materials and Methods

### Biological materials

The HEK 293T cell line was obtained from J.C. de la Torre and the HeLa cell line was acquired from ATCC. They were cultivated as described [31]. The vaccinia virus vTF7-3 [32] was a gift of B. Moss. Plasmids pGPB1, pGPB2, pGPA and pGNP have been described [19]. Plasmids pGPB1TAP and pGPB2TAP, with the TAP tag [29] fused to the C-terminus of either PB1 or PB2 were described previously [33]. The ORFs of PB1, PB2 and PA were transferred from the pG plasmid series [19] to pcDNA3.1 to generate plasmids pCPB1, pCPB2 and pCPA. Plasmid pCHisTEVPA was constructed by insertion of a 6xHis tag and the TEV cleavage site at the N-terminus of PA (sequences available on request). The PB2 SV mutant series was generated by insertion of a SV40 TAg NLS sequence at the C-terminus of the protein (sequences available on request). Plasmid pCMVNP has been described [34]. The inserts of plasmids pT7NSCAT-RT and pT7ΔNSclone23 [18] were transferred to pHH21 [35] to generate plasmids pHHNSCAT and pHHclone23. Plasmids containing the cDNAs of PB2 from A/Goose/Guandong/1/97 (H5N1), A/Thailand/Kan1/04 (H5N1) and A/Vietnam/1203/04 (H5N1) were obtained from S. Pleschka, G. Gabriel and R. Donis, respectively. Their ORFs were transferred to pGPB2TAP to generate plasmids pGPB2GGuaTAP, pGPB2ThTAP and pGPB2VnTAP, respectively.

Anti-PB1 serum and anti-PB2 monoclonal antibodies have been described [18], [36], [37]. Rabbit antisera specific for NP was generated by immunisation with purified His-NP. Antibodies specific for α5-importin, α1-importin and α5/α7-importin were purchased to Abnova, BD Biosciences and Sigma, respectively.

### Biochemical analyses

To purify PB2 associated proteins, HEK 293T cells were infected with vTF7-3 virus at a moi of 10 pfu/cell and transfected with plasmid pGPB2TAP, or pGPB2 as a control, using the calcium phosphate transfection protocol [38]. After 16–20 h incubation at 37°C, extracts were prepared and the PB2-containing complexes were purified by the TAP approach [33]. Western-blotting and silver-staining were carried out as described [33], [39]. For immunofluorescence, HeLa cells were transfected with pCPB2 plasmid or mutants thereof using Fugene HD reagent (GE Healthcare) as recommended by the manufacturer and fixed with 3,7% formaldehyde at 20 hours post-transfection. HEK293T cells were transfected with PB2 alone or with the three subunits of the polymerase as indicated above. At 16 hours post-transfection they were seeded on coverslips and fixed 16 hours thereafter. The cells were permeabilised with 0.5% Triton ×100 and processed for indirect immunofluorescence with anti-PB2 monoclonal or anti-PB1 polyclonal antibodies as described before [40]. Images were collected on a Leica SP5 confocal microscope (Leica Microsystems) and analized with the LAS AF Software (Leica Microsystems).

### Polymerase and RNP activity

To assay polymerase complex formation, HEK293T cell cultures were co-transfected with plasmids pCPB1, pCPB2 (or mutants thereof) and pCHisTEVPA. Total cell extracts were analysed by ^2+^Ni-NTA-agarose chromatography as described [5] and Western-blot using anti-PB2 antibodies. To determine the RNP biological activity (transcription plus replication), HEK293T cells were co-transfected with plasmids pCPB1, pCPB2 (or mutants thereof), pCPA, pCMVNP and pHHNSCAT as indicated. At 20 h post-transfection, total cell extracts were prepared and CAT protein was determined by ELISA (GE Healthcare). The replication of recombinant RNPs was determined as follows: HEK293T cells were infected with vaccinia vTF7-3 and transfected with plasmids pGPB1TAP or pGPB1, pGPA, pGNP, pT7ΔNS-RT clone 23 and either pCPB2 or mutants thereof [4], [18]. At 24 hours post-transfection, extracts were prepared and the RNPs amplified in vivo were purified by binding to IgG-Sepharose and cleavage with TEV protease [33]. The accumulation of progenie RNPs was determined by Western-blot using anti-NP sera. The activity of the purified RNPs was tested by ApG- and β-globin mRNA-driven in vitro transcription [5], [41].

### Proteomic techniques

PB2 associated proteins were separated by PAGE and stained with PAGE-blue ™ (Fermentas). Proteins of interest were excissed manually and processed as described [16]. The resulting tryptic peptides were prepared using a standard dried droplet technique [42] by overlaying the sample and matrix solutions, spotting an aliquot of 0.8 µl of eluted peptides onto a 384-well Opti-TOF ™ plate (Applied Biosystems, Framingham, MA) and air drying the samples. The same volume of matrix solution (α-cyano-4-hydroxi cinnaminic acid in 30% acetonitrile-15% isopropanol-0.5% TFA) was deposited.

Peptide mass fingerprinting for each protein, and subsequent MS/MS analysis, were obtained automatically in a 4800 MALDI-TOF/TOF™ Analyzer (Applied Biosystems, Framingham, MA), first calibrated externally. For acquisition and peak lists generation of each spectrum, the 4000 Series Explorer software v3.5.28193 (Applied Biosystems) was used. For protein identification, the combined peptide mass fingerprint and MS/MS data search was performed using GPS Explorer™ software v3.6 (Applied Biosystems) against the NCBInr database using MASCOT 2.1 software (Matrix Science, London, UK). Search parameters were: carbamidomethyl cystein as fixed modification by the treatment with iodoacetamide, oxidized methionines as variable modification and 1 missed cleavage site allowed. Peptide and fragment mass tolerance were set to 100 ppm and ±0.8 Da, respectively. In all protein identifications, the probability protein and peptide mowse scores were greater than the minimum score fixed as significant.

## Supporting Information

Figure S1 Expression of wt and mutant PB2 proteins. Cultures of HEK293T cells were transfected with plasmids expressing wt PB2 (WT), mutant PB2, with and without added TAg NLS at their C-terminus, or mock-transfected (No PB2). Total cell extracts were prepared and analysed by Western-blot using monoclonal antibodies specific for PB2. An extract obtained from influenza virus-infected cells was used as mobility marker (Flu).
(1.66 MB TIF)

Figure S2 Intracellular localisation of wt and mutant PB2 proteins. Cultures of HEK293T cells were transfected with plasmids expressing wt PB2, mutant PB2 or mock-transfected. The localisation of PB2 was analysed by immunofluorescence using monoclonal antibodies specific for PB2. Central optical sections are presented of HEK293T cells either mock-transfected (MOCK), transfected with wt PB2 (WT) or with each of the mutant PB2 proteins indicated. Nuclei were stained with DAPI (blue) and PB2 was stained with anti-PB2 monoclonal antibody and goat anti-mouse IgG coupled with Alexa 488 (green).
(5.40 MB TIF)

Table S1 Summary of proteomic identifications.
(0.03 MB DOC)

Table S2 Complete data for the proteomic analysis.
(0.13 MB DOC)
