# Supplementary figures and images for: The Host-Dependent Interaction of α-Importins with Influenza PB2 Polymerase Subunit Is Required for Virus RNA Replication
Source: PLoS One. 2008 Dec 10;3(12):e3904. doi: 10.1371/journal.pone.0003904 (PMC2588535; doi:10.1371/journal.pone.0003904)

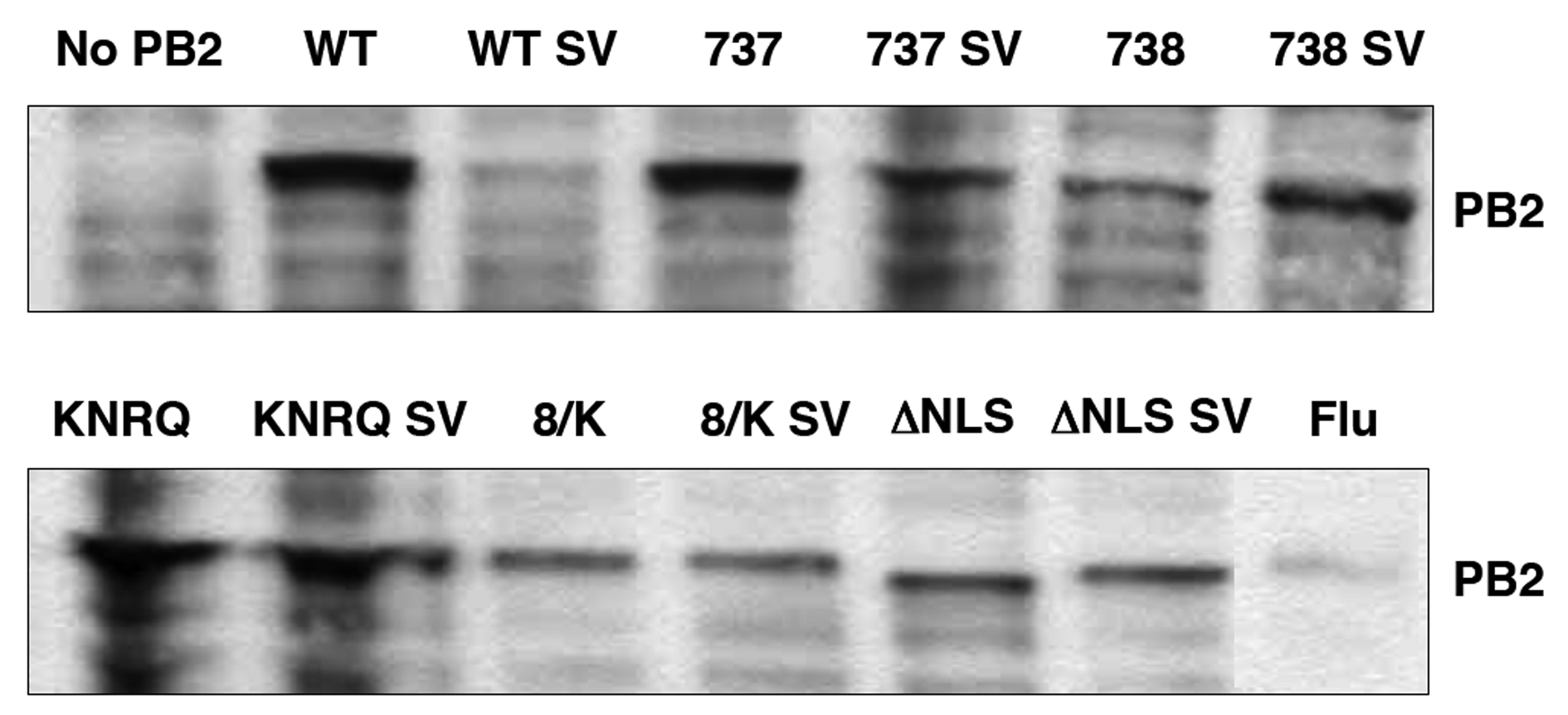

Supplement: Figure S1 — Expression of wt and mutant PB2 proteins. Cultures of HEK293T cells were transfected with plasmids expressing wt PB2 (WT), mutant PB2, with and without added TAg NLS at their C-terminus, or mock-transfected (No PB2). Total cell extracts were prepared and analysed by Western-blot using monoclonal antibodies specific for PB2. An extract obtained from influenza virus-infected cells was used as mobility marker (Flu). (1.66 MB TIF) [file pone.0003904.s001.tif]

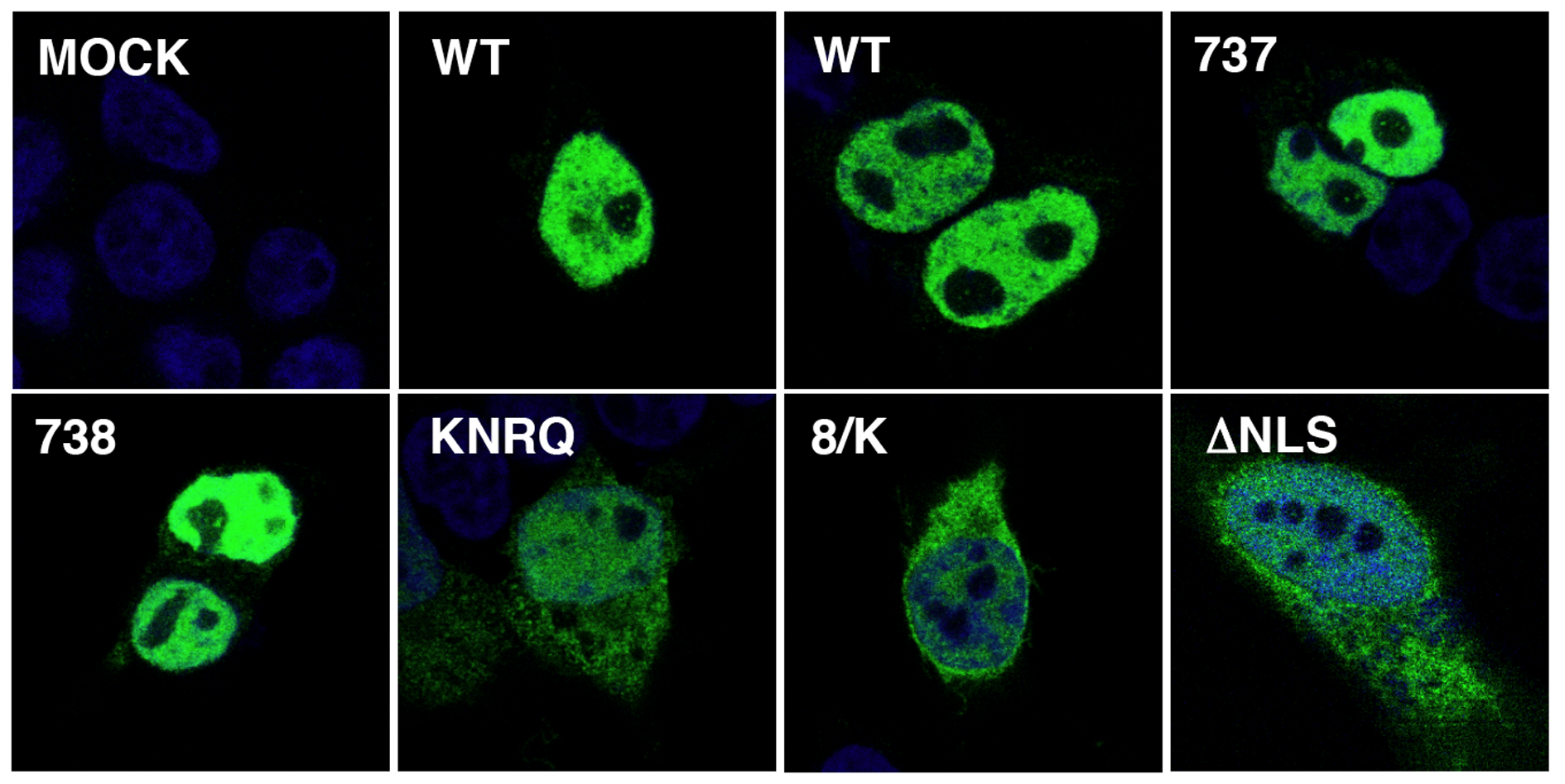

Supplement: Figure S2 — Intracellular localisation of wt and mutant PB2 proteins. Cultures of HEK293T cells were transfected with plasmids expressing wt PB2, mutant PB2 or mock-transfected. The localisation of PB2 was analysed by immunofluorescence using monoclonal antibodies specific for PB2. Central optical sections are presented of HEK293T cells either mock-transfected (MOCK), transfected with wt PB2 (WT) or with each of the mutant PB2 proteins indicated. Nuclei were stained with DAPI (blue) and PB2 was stained with anti-PB2 monoclonal antibody and goat anti-mouse IgG coupled with Alexa 488 (green). (5.40 MB TIF) [file pone.0003904.s002.tif]
