# Supplementary material for: The Host-Dependent Interaction of α-Importins with Influenza PB2 Polymerase Subunit Is Required for Virus RNA Replication
Source: PLoS One. 2008 Dec 10;3(12):e3904. doi: 10.1371/journal.pone.0003904 (PMC2588535; doi:10.1371/journal.pone.0003904)
Supplement: Table S1 — Summary of proteomic identifications. (0.03 MB DOC) [file pone.0003904.s003.doc]

| **Accession. Number^a^** | **Protein Name** | **Mr^b^** | **MASCOT Score^c^** | **Nº matched peptides** | **Sequence coverage** | **Nº fragmented peptides** | **p-Values** |
| --- | --- | --- | --- | --- | --- | --- | --- |
| gi|401030 | Polymerase basic protein 2  (RNA-directed RNA polymerase subunit P3) | 86356 | 337 | 10 | 15% | 7 | 1.1e-027 |
| gi|60552566 | Karyopherin alpha 1 (importin alpha 5) | 60955 | 80 | 5 | 11% | 2 | 0.0019 |

^a^ Accession number in NCBInr protein database.

^b^ Theoretical nominal mass.

^c^ MASCOT score is the result of combining peptide mass fingerprint spectra with the fragmentation data of several peptides.
