# Supplementary material for: The Host-Dependent Interaction of α-Importins with Influenza PB2 Polymerase Subunit Is Required for Virus RNA Replication
Source: PLoS One. 2008 Dec 10;3(12):e3904. doi: 10.1371/journal.pone.0003904 (PMC2588535; doi:10.1371/journal.pone.0003904)
Supplement: Table S2 — Complete data for the proteomic analysis. (0.13 MB DOC) [file pone.0003904.s004.doc]

|  | |  | | | | | |  | | | |  | | | |  | |  | | | | |  | | | | | |  |  | |
| --- | --- | --- | --- | --- | --- | --- | --- | --- | --- | --- | --- | --- | --- | --- | --- | --- | --- | --- | --- | --- | --- | --- | --- | --- | --- | --- | --- | --- | --- | --- | --- |
| [**Taxonomy: Influenza A virus (A/Victoria/3/1975(H3N2))**](http://www.ncbi.nlm.nih.gov/htbin-post/Taxonomy/wgetorg?lvl=0&lin=f&id=392809) | | | | | | | | | | | | | | | | | | |  | | |  | |  |  |  |  |  | | |  |
| Match to: **gi|401030** Score: **337** Expect: **1.1e-027** | | | | | | | | | | | | | | | | | | | |  |  |  | |  |  |  |  |  | | |  |
| Nominal mass (M_r_): **86356**; Calculated pI value: **9.59** | | | | | | | | | | | | | | | | | | |  | | |  | |  |  |  |  |  | | |  |
| Sequence Coverage: **15%** | | |  | | |  | | |  | |  | | | | | | Observed | | | | | Delta | | Start | End | Miss | Ion scoreª | Peptide | | |  |
|  |  | | |  |  | |  | |  | |  | | | | | | **913,45** | | | | | 0 | | 590 | 597 | 0 | **13** | **R.GQYSGFVR.T** | | |  |
| **1** MERIKELRNL MSQSRTREIL TKTTVDHMAI IKKYTSGRQE KNPSLRMKWM | | | | | | | | | | | | | | | | | 913,45 | | | | | 0 | | 590 | 597 | 0 | --- | R.GQYSGFVR.T | | |  |
| **51** MAMKYPITAD KRITEMVPER NEQGQTLWSK MSDAGSDRVM VSPLAVTWWN | | | | | | | | | | | | | | | | | 1137,6 | | | | | -0,02 | | 428 | 436 | 0 | --- | R.LNPMHQLLR.H + Oxidation (M) | | | |
| **101** RNGPVTSTVH YPKVYKTYFD KVER**LKHGTF GPVHFR**NQVK IRRRVDINPG | | | | | | | | | | | | | | | | | 1154,57 | | | | | -0,02 | | 127 | 136 | 0 | --- | K.HGTFGPVHFR.N | | |  |
| **151** HADLSAK**EAQ DVIMEVVFPN EVGAR**ILTSE SQLTITKEKK EELQDCKISP | | | | | | | | | | | | | | | | | **1395,74** | | | | | -0,02 | | 125 | 136 | 1 | **16** | **R.LKHGTFGPVHFR.N** | | |  |
| **201** LMVAYMLERE LVRKTRFLPV AGGTSSVYIE VLHLTQGTCW EQMYTPGGEV | | | | | | | | | | | | | | | | | 1395,74 | | | | | -0,02 | | 125 | 136 | 1 | --- | R.LKHGTFGPVHFR.N | | |  |
| **251** R**NDDIDQSLI IAAR**NIVRRA SVSADPLASL LEMCHSTQIG GTRMVDILRQ | | | | | | | | | | | | | | | | | **1443,72** | | | | | -0,03 | | 252 | 264 | 0 | **57** | **R.NDDIDQSLIIAAR.N** | | |  |
| **301** NPTEEQAVDI CKAAMGLRIS SSFSFGGFTF KRTSGSSIK**R EEEVLTGNLQ** | | | | | | | | | | | | | | | | | 1443,72 | | | | | -0,03 | | 252 | 264 | 0 | --- | R.NDDIDQSLIIAAR.N | | |  |
| **351 TLK**IRVHEGY EEFTMVGKRA TAILRKATRR LVQLIVSGRD EQSIAEAIIV | | | | | | | | | | | | | | | | | 1467,69 | | | | | -0,03 | | 575 | 586 | 0 | **17** | K.MEFEPFQSLVPK.A + Oxidation (M) | | | |
| **401** AMVFSQEDCM IKAVRGDLNF VNRANQR**LNP MHQLLR**HFQK DAKVLFQNWG | | | | | | | | | | | | | | | | | 1467,69 | | | | | -0,03 | | 575 | 586 | 0 | --- | K.MEFEPFQSLVPK.A + Oxidation (M) | | | |
| **451** IEHIDNVMGM VGVLPDMTPS TEMSMRGIRV SKMGVDEYSS TERVVVSIDR | | | | | | | | | | | | | | | | | **1589,81** | | | | | -0,05 | | 704 | 718 | 0 | **50** | **R.YGPALSINELSNLAK.G** | | |  |
| **501** FLRVRDQR**GN VLLSPEEVSE THGTER**LTIT YSSSMMWEIN GPESVLVNTY | | | | | | | | | | | | | | | | | 1589,81 | | | | | -0,05 | | 704 | 718 | 0 | --- | R.YGPALSINELSNLAK.G | | |  |
| **551** QWIIRNWETV KIQWSQNPTM LYNK**MEFEPF QSLVPK**AIR**G QYSGFVR**TLF | | | | | | | | | | | | | | | | | 1629,86 | | | | | -0,02 | | 340 | 353 | 1 | --- | K.REEEVLTGNLQTLK.I | | |  |
| **601** QQMRDVLGTF DTTQIIKLLP FAAAPPKQSR MQFSSLTVNV RGSGMRILVR | | | | | | | | | | | | | | | | | **1953,93** | | | | | -0,02 | | 509 | 526 | 0 | **55** | **R.GNVLLSPEEVSETHGTER.L** | | |  |
| **651** GNSPVFNYNK TTKRLTILGK DAGTLIEDPD ESTSGVESAV LRGFLILGKE | | | | | | | | | | | | | | | | | 1953,93 | | | | | -0,02 | | 509 | 526 | 0 | --- | R.GNVLLSPEEVSETHGTER.L | | |  |
| **701** DRR**YGPALSI NELSNLAK**GE KANVLIGQGD VVLVMKRKRD SSILTDSQTA | | | | | | | | | | | | | | | | | **2002,98** | | | | | -0,01 | | 158 | 175 | 0 | **82** | **K.EAQDVIMEVVFPNEVGAR.I** | | |  |
| **751** TKRIRMAIN | | | |  | | | | | |  | | |  |  |  | | 2002,98 | | | | | -0,01 | | 158 | 175 | 0 | --- | K.EAQDVIMEVVFPNEVGAR.I | | |  |
|  |  | | |  | | | | | |  | | |  |  |  | |  | | | | |  | |  |  |  |  |  | | |  |
|  |  | | |  | | | | | |  | | |  |  |  | |  | | | | |  | |  |  |  |  |  | | |  |
|  |  | | |  | | | | | |  | | |  |  |  | | ª Sequenced peptides and the corresponding ion score are shown in bold type | | | | | | | | | | | | | |  |

| **karyopherin alpha 1 [Homo sapiens]** | | | | | | | | | |  | | | |  |  |  |  |  |  |  |  |
| --- | --- | --- | --- | --- | --- | --- | --- | --- | --- | --- | --- | --- | --- | --- | --- | --- | --- | --- | --- | --- | --- |
| [**Taxonomy: Homo sapiens**](http://www.ncbi.nlm.nih.gov/htbin-post/Taxonomy/wgetorg?lvl=0&lin=f&id=9606) | | | | | |  | |  | |  | | | |  |  |  |  |  |  |  |  |
| Match to: **gi|88758611** Score: **80** Expect: **0.0019** | | | | | | | | | | | | | |  |  |  |  |  |  |  |  |
| Nominal mass (M_r_): **60952**; Calculated pI value: **4.94** | | | | | | | | | | | |  |  | | | |  |  |  |  |  |
| Sequence Coverage: **11%** | | |  | |  | |  | |  | | |  |  | | | |  |  |  |  |  |
|  |  |  | |  |  | |  | |  | | |  |  | | | |  |  |  |  |  |
| **1** MTTPGKENFR LKSYKNKSLN PDEMRRRREE EGLQLRKQKR EEQLFKRRNV | | | | | | | | | | | |  |  | | | |  |  |  |  |  |
| **51** ATAEEETEEE VMSDGGFHEA QINNMEMAPG GVITSDMIEM IFSKSPEQQL | | | | | | | | | | | | Observed | Delta | | | | Start | End | Miss | Ion scoreª | Peptide |
| **101** SATQKFRKLL SK**EPNPPIDE VISTPGVVAR** FVEFLKRKEN CTLQFESAWV | | | | | | | | | | | | 1303,66 | 0 | | | | 231 | 241 | 0 | --- | R.NAVWALSNLCR.G |
| **151** LTNIASGNSL QTRIVIQAGA VPIFIELLSS EFEDVQEQAV WALGNIAGDS | | | | | | | | | | | | 1488,78 | 0 | | | | 231 | 243 | 1 | --- | R.NAVWALSNLCRGK.S |
| **201** TMCRDYVLDC NILPPLLQLF SKQNRLTMTR **NAVWALSNLC RGK**SPPPEFA | | | | | | | | | | | | **1551,91** | -0,01 | | | | 439 | 452 | 0 | **29** | **K.IVQVALNGLENILR.L** |
| **251** KVSPCLNVLS WLLFVSDTDV LADACWALSY LSDGPNDKIQ AVIDAGVCRR | | | | | | | | | | | | 1551,91 | -0,01 | | | | 439 | 452 | 0 | --- | K.IVQVALNGLENILR.L |
| **301** LVELLMHNDY KVVSPALRAV GNIVTGDDIQ TQVILNCSAL QSLLHLLSSP | | | | | | | | | | | | **1889,98** | -0,01 | | | | 113 | 130 | 0 | **30** | **K.EPNPPIDEVISTPGVVAR.F** |
| **351** KESIKKEACW TISNITAGNR AQIQTVIDAN IFPALISILQ TAEFRTRKEA | | | | | | | | | | | | 1889,98 | -0,01 | | | | 113 | 130 | 0 | --- | K.EPNPPIDEVISTPGVVAR.F |
| **401** AWAITNATSG GSAEQIKYLV ELGCIKPLCD LLTVMDSK**IV QVALNGLENI** | | | | | | | | | | | | 1905,9 | -0,03 | | | | 481 | 495 | 0 | --- | K.IEFLQSHENQEIYQK.A |
| **451 LR**LGEQEAKR NGTGINPYCA LIEEAYGLDK **IEFLQSHENQ EIYQK**AFDLI | | | | | | | | | | | |  |  | | | |  |  |  |  |  |
| **501** EHYFGTEDED SSIAPQVDLN QQQYIFQQCE APMEGFQL | | | | | | | | | | | | | |  |  |  |  |  |  |  |  |
|  |  |  | | | |  | |  | | |  | | |  |  |  |  |  |  |  |  |
|  |  |  | | | |  | |  | | |  | | |  | ª Sequenced peptides and the corresponding ion score are shown in bold type | | | | | | |
